# Short communication: salivary cortisol concentrations and lying behavior of ewes in response to semi-laparoscopic and laparoscopic embryo transfer

**DOI:** 10.1093/jas/skaf381

**Published:** 2025-11-06

**Authors:** Melinda Bagi, Viktor Jurkovich, Levente Kovács, Szilárd Bodó, Mikolt Bakony, János Oláh, László Huzsvai, Nóra Vass

**Affiliations:** Department of Animal Husbandry, Faculty of Agricultural and Food Sciences and Environmental Management, Institute of Animal Science, Biotechnology and Nature Conservation, University of Debrecen, Debrecen, Hungary; Centre for Animal Welfare, University of Veterinary Medicine, Budapest, Hungary; Department of Animal Husbandry and Animal Welfare, Institute of Animal Sciences, Hungarian University of Agriculture and Life Sciences Institute of Animal Science, Gödöllő, Hungary; Department of Precision Livestock Farming and Animal Biotechnics, Hungarian University of Agriculture and Life Sciences, Institute of Animal Science, Kaposvár, Hungary; Centre for Translational Medicine, Semmelweis University, Budapest, Hungary; Department of Animal Husbandry, Faculty of Agricultural and Food Sciences and Environmental Management, Institute of Animal Science, Biotechnology and Nature Conservation, University of Debrecen, Debrecen, Hungary; Department of Quantitative Methodology, Faculty of Economics and Business, Institute for Methodology and Business Digitalisation, University of Debrecen, Debrecen, Hungary; Department of Animal Husbandry, Faculty of Agricultural and Food Sciences and Environmental Management, Institute of Animal Science, Biotechnology and Nature Conservation, University of Debrecen, Debrecen, Hungary

**Keywords:** animal welfare, embryo transfer, lying behavior, saliva cortisol, stress

## Abstract

This study compared the short-term effects of the laparoscopic and semi-laparoscopic embryo transfer (**ET**) procedures on salivary cortisol concentrations and the lying behavior of ewes. In total, 40 ewes were synchronized for ET and placed randomly into individual pens 2 d before the operations. On the day of the operations, laparoscopic (**L**, *n* = 15) and semi-laparoscopic (**SL**, *n* = 10) ET were performed. At the same time, control animals (**Control**, *n* = 15) were placed into the laparoscopic cradle for 6 min without an ET procedure. Monitoring of standing and lying behaviors started 24 h before the surgery and lasted until 24 h after the procedures were completed. Saliva samples were taken 4 times during the experiment and assayed for cortisol concentrations. Saliva cortisol concentrations were elevated in all groups 1 (Control: 4.8 ± 0.5 ng/mL; L: 5.1 ± 0.5 ng/mL; SL: 4.2 ± 0.7 ng/mL) and 2 (Control: 5.5 ± 0.5 ng/mL; L: 5.9 ± 0.5 ng/mL; SL: 4.8 ± 0.7 mg/mL) hours after the procedures compared to the initial concentration (Control: 3.8 ± 0.5 ng/mL; L: 3.7 ± 0.5 ng/mL; SL: 3.1 ± 0.7 ng/mL), and returned to the basal levels for the end of the 24-h post-surgery period (Control: 3.4 ± 0.5 ng/mL; L: 4.1 ± 0.5 ng/mL; SL: 3.5 ± 0.7 ng/mL). There were no significant differences in the cortisol levels among the groups. There was no difference in the total lying time between the groups 24 h before or after the surgery. The average length of lying bouts calculated for the 24-h post-surgery period increased in all groups from the 24-h pre-surgery period, with a parallel decrement in the number of lying bouts (*P* < 0.05). Our results suggest that the laparoscopic and semi-laparoscopic ET did not cause more stress in the ewes than the handling procedures related to surgery and preparation.

## Teaser

The laparoscopic and semi-laparoscopic embryo transfer procedures did not cause considerable stress to the ewes.

## Introduction

Amidst the growing societal concern for ethical issues and the importance of animal welfare in agriculture, the demand for welfare-friendly products is rising (Steagall et al., 2021). In this context, veterinarians are responsible for selecting appropriate interventions, managing pain, and designing treatment methods and protocols from an animal welfare perspective (Steagall et al., 2021; Hernandez et al., 2022).

Assisted reproductive technologies (**ART**) such as artificial insemination (**AI**) and embryo transfer (**ET**) are frequently used in the sheep industry to ensure genetic improvement and reproductive performance (Amiridis and Cseh, 2012; Figueira et al., 2020). In the multiple ovulation and embryo transfer (**MOET**) programs, the donor ewes are synchronized and superovulated before AI and embryo collection (Figueira et al., 2020). The embryos are collected 7–8 d after AI, primarily either by surgical (mid-ventral laparotomy) or minimally invasive laparoscopic techniques (Naitana and Ledda, 2020). The nonsurgical (transcervical) technique can also be performed, but this method has limitations, such as preparations related to uterine flushing, chemical restraint, cervical transposing (Fonseca et al., 2016). For ET, semi-laparoscopic (**SL**) or laparoscopic (**L**) techniques are used primarily under field conditions. The transcervical technique is not widely used for ET, mainly due to the anatomical structure of the cervix of ewes (McMillan and Hall, 1994; McKelvey, 1999; Gibbons and Cueto, 2022). The average success rate of the various methods is 34–80% for SL (McMillan and Hall, 1994), 37–80% for L (Santos et al., 2020), and 33% for the transcervical (Fonseca et al., 2016). Some methods (such as surgical or L) require at least 20–24 h of fasting and 12 h without water, mainly to avoid anesthetic complications and prevent organ injury (Santos et al., 2020). During the transcervical technique, fasting is required only when the animals are in dorsal recumbency, not in a standing position. The nonsurgical method can prevent adhesions in the abdominal cavity. However, the transcervical technique can also cause stress and damage by the manipulation of the cervix (Fonseca et al., 2016; Santos et al., 2020).

The behavioral and physiological responses to distress related to postoperative pain are increasingly studied in sheep (Silva et al., 2020). An essential indicator of stress is the cortisol level in different media, such as saliva, blood, urine, feces, or hair (Palme, 2012). Collecting saliva samples is a less invasive method for measuring cortisol levels than blood sampling (Palme, 2012). It has been successfully used in sheep (Andanson et al., 2020), goats (Greenwood and Shutt, 1992), and cattle (Kovács et al., 2016).

Accelerometers are frequently used in behavioral studies on cattle, pigs, and small ruminants to measure, for instance, time spent lying, standing, and walking (Caja et al., 2020; Chapa et al., 2020). HOBO Pendant G accelerometers were validated for cattle (Bonk et al., 2013) and, more recently, for small ruminants (Radeski and Ilieski, 2017; Williams et al., 2021). The use of accelerometers in sheep and goat behavioral studies is continuously growing to measure grazing (Kampherbeek et al., 2023), to display lying and general behavioral patterns (Barwick et al., 2018a), and to predict parturition (Fogarty et al., 2020). Besides measuring normal behavior, accelerometers are efficient in recognizing the behavioral changes associated with lameness (Barwick et al., 2018b) or other types of illness (Gurule et al., 2022).

Our study aimed to compare the effects of L and SL ET techniques on saliva cortisol concentrations and lying behavior as non-invasive approaches to stress in sheep. We expected lower levels of saliva cortisol and unchanged lying behavior during the investigation of post-surgery stress in L ewes compared to SL ewes.

## Materials and Methods

### Animals, feeding, and housing

The study was approved by the Department of Food Chain Safety and Animal Health at Hajdú-Bihar County Governmental Office (Permit Number: 33-M/2022/DEMÁB). The experiment was performed in accordance with the relevant guidelines and regulations on animal welfare.

The study was carried out in Debrecen, Hungary, at the Research Farm of the University of Debrecen between the 23rd and 27th of April 2023. The Research farm has two neighboring units with similar housing conditions. Forty crossbred meat-type ewes, aged 2–6 yr, with body condition scores between 2.5 and 3.5, were selected from the two units for the study. They were kept in semi-intensive conditions, on a closed pasture during the day, in a pen in the evening, with ad libitum access to water and hay. Two months before the onset of the study, they were kept only in a closed pen. Feed was distributed twice daily. The animals were placed in 2 × 1.5 m individual pens 2 d before the ART operations to help them habituate to the new environment and to avoid disturbing ewes while handling their herdmates during the experiment. The pens were arranged in three rows, allowing the animals to be in physical contact with their neighbors and to have visual and auditory contact with the others. Ad libitum access to feed and water was offered on the first day of separation. Feed and water were withdrawn from the animals 24 h before the operation to minimize the possibility of regurgitation and to allow easier visual observation of the reproductive tract. The animals were taken to the operating room individually on the day of the operation. After the ART procedures, the ewes were returned to their individual pens for a 24-h recovery and observation period, during which they had free access to feed and water.

### Embryo transfer procedures

The ewes were randomly assigned into three groups (Control: *n* = 15; L: *n* = 15; SL: *n* = 10). They were treated on two consecutive days, evenly distributed. Heat synchronization of the ewes was achieved by inserting Chronogest (MSD Animal Health, France) intravaginal sponges for 14 d. On the day of the sponge withdrawal, 300 IU eCG (Folligon inj. AU.V., MSD Animal Health, France) was injected intramuscularly. The L or SL ET was carried out 6 d after the sponge withdrawal. The L recipients were anesthetized with 40–50 µg/kg detomidine (Domosedan inj. A.U.V., Orion, Finland) and laid in dorsal recumbency in a special operation cradle (Stafford et al., 2006). The caudal part of the cradle is elevated at 45°, ensuring the Trendelenburg position. In the L ewes, two 1 cm incisions were made approximately 5 cm from the udder and 5 cm from the midline on both sides. After the two trocars were inserted into the abdominal cavity, the corpus luteum was checked using the laparoscopic optic (Karl Storz SE & Co. KG, Tuttlingen, Germany), and the embryo was transferred into the uterine horn with an Aspic insemination catheter (IMV Technologies, France). In the SL ewes, a combination of 20 µg/kg detomidine (Domosedan inj. A.U.V., Orion Oyj, Finland) and 2 mg/kg ketamine (Ketamidor 100 mg/ml inj. A.U.V., Richter Pharma AG, Austria) was used intramuscularly for anesthesia (Ewing, 1990). The operation technique was the same as written in L, but after examining the ovaries, the ipsilateral uterine horn was clamped with atraumatic forceps. One of the incisions was extended to 2–3 cm, and the uterine horn was pulled out from the abdominal cavity; the embryo was inserted with an embryo catheter (IMV Technologies, France), and the uterus was allowed to slide back. The incisions were closed with sutures. When finishing the operation, the animals were treated with 15 mg/kg amoxicillin (Betamox L.A. inj. A.U.V., Norbrook, UK) as an antibiotic and 1 mg/kg meloxicam (Melovem inj. A.U.V., Dopharma Research B.V., the Netherlands) as a nonsteroidal anti-inflammatory drug (Cohen et al., 2024). Control ewes were subjected to a sham operation, meaning that C animals were also anesthetized and placed into the operation cradle in the Trendelenburg position, where they spent 6 min without surgery. The C animals were taken back to their pen after the sham operation procedure. The duration of the L and SL operations was, on average, 6.6 and 8.3 min, respectively.

### Saliva sampling and analysis

Saliva samples were collected from the animals with synthetic swabs (Sailvette Cortisol, Sarstedt GmbH, Germany) four times during the experimental period. The first samples were collected from each ewe on the morning of the operation. The second and third samples were taken 1 and 2 h after the animals returned to their pens (after the surgery). The fourth sample was collected 24 h after the surgery. The sampling rolls were held with Collin tongue forceps and then put into the sheep’s mouths between the molars and buccal area when the sheep were not ruminating to avoid sample contamination. The sheep were allowed to chew on the rolls for approximately 20 s for proper saliva collection. The Salivettes were immediately cooled to 4 °C and transported to the laboratory. The swabs were centrifuged at 3000 *g* for 10 min, and the saliva (at least 1.5 mL per sample) was stored at –80 °C in Eppendorf tubes until the assay. A direct homemade RIA method was used for cortisol analysis, developed for determining cortisol in the plasma of food-producing animals using 1,2,6,7–3H-cortisol (TRK 407; Radiochemical Center, UK) and a highly specific polyclonal antibody raised against cortisol-21-HS-BSA in rabbits (Csernus, 1982). Cross-reactivity of the assay: cortisol: 100%; corticosterone: 19%; prednisolone: 9.5%; deoxycortisol: 6.4%; 17α-OH progesterone: 5.7%; progesterone: 2.6%; any other 22 steroids: 0.54 to 0.0001%. The assay standards (cortisol FW 362.5; Sigma Chemical Company, USA) were prepared in cortisol-free plasma. The antibody-bound and the free fractions were separated by cold dextran-coated charcoal suspension after an 18 to 24-h incubation period. Radioactivity was measured by a TriCarb liquid scintillation counter (Perkin Elmer Inc., Downers Grove, IL, USA). The sensitivity of this assay system was 11.37 fmol/tube. Within the concentration range of about 2.0 and 100.0 nmol/mL, the intra- and interassay coefficients of variation varied between 3 and 8% and 5 and 10%, respectively. Samples with cortisol concentrations higher than 100.0 nmol/mL were re-assayed after dilution (Csernus, 1982).

### Assessment of lying behavior

Accelerometers (HOBO Pendant G logger, Onset Computer Corp., USA) were fitted to the left hind leg of the animals on the outside of the midfoot, and cotton wool was placed underneath and secured with a Copoly flexible cohesion bandage (M + H Vet Ltd, Czech Republic). For proper habituation, the accelerometers were placed on the animals’ legs on the day they were placed in the individual pens, 2 d before the ART operations. The device measured and recorded the *g*-force and tilt on the *x*, *y*, and *z*-axes on a scale of −1 to 1 (x, y, z) and was suitable for separating the standing and lying postures. Sampling was set at 30-s intervals, and cut-off values of *g *< 0.75 for lying down and g ≥ 0.75 for standing were determined using the x-axis measurements. These values were used to categorize logger readings as standing or lying (Bonk et al., 2013). Forty-eight hours after surgery, the device was removed, and data were downloaded and exported to Excel. Output was edited to examine the effect of using a 1-min event filter to remove potentially erroneous readings of lying or standing events (Bonk et al., 2013). The first hour after the procedure was excluded from analyses due to the recovery phase from anesthesia. Total time spent lying, the number of episodes of lying down, the average length of a lying bout, and the minimum and maximum length of lying bouts were determined for the 24 h before and 24 h after surgery.

### Statistical analysis

Following the graphical exploration of data distribution and association patterns, the least squares means of parameter values were compared between groups and across sampling times. Linear mixed models were fitted with untransformed least squares means of the studied animal-based parameters as the response parameter and group, sampling time, and their interaction term as predictors. Sheep ID nested in the farm unit was included as a random term. Model fit was evaluated by checking the normality of random effects using quantile-quantile plots. Effects of treatment and time were evaluated with post-hoc tests comparing differences of estimated marginal means between different sampling times and groups. *P*-values were adjusted for multiple comparisons using the Bonferroni-Holm method. Interaction terms were not removed from models, even when they were non-significant and included in model-based estimations. The level of significance was set at *P* < 0.05. All data analyses were performed using the R statistical software (version 4.4.1; R Core Team, 2021). The latest versions of the packages “ggplot2,” “nlme,” and “emmeans” were used for analysis.

## Results

### Saliva cortisol concentration

The main effect of treatment and the interaction term for group x sampling time were not significant (*P* = 0.4113 and *P* = 0.9772, respectively). However, sampling time was significantly associated with cortisol concentrations (*P* < 0.0001). Saliva cortisol concentrations (mean and standard errors) according to treatment groups and sampling times are shown in Table 1. In all groups, cortisol levels were numerically, but not statistically significantly, higher 1 h after surgery. The highest concentrations were measured at the 2 h post-surgery sampling in all treatments; however, only the values of the control and laparoscopic groups differed significantly from baseline. Twenty-four hours after surgery, cortisol concentrations were not different from baseline in any of the groups.

**Table 1.** Saliva cortisol concentrations according to treatment and sampling time (model-based mean ± SE)

Cortisol concentration (ng/ml)	Baseline	1 h after surgery	2 h after surgery	24 h after surgery
**Control**	3.8 ± 0.5^a^	4.8 ± 0.5^ab^	5.5 ± 0.5^b^	3.4 ± 0.5^a^
**Laparoscopy**	3.7 ± 0.5^a^	5.1 ± 0.5^ab^	5.9 ± 0.5^b^	4.1 ± 0.5^a^
**Semi-laparoscopy**	3.1 ± 0.7	4.2 ± 0.7	4.8 ± 0.7	3.5 ± 0.7

Different letters in superscripts indicate significant differences within a row. Different numbers in superscripts indicate significant differences within a column. The absence of superscripts indicates no significant difference.

### Lying behaviour

Model-based estimates for measures of lying behavior are displayed in Table 2. For *total lying time*, no significant interaction was found between treatment and time (*P* = 0.8600). Although the F-test revealed a significant association between treatment and outcome (*P* = 0.0382), corrections for multiple comparisons increased the *P*-values, so no difference between groups could be detected. Sampling time was also not significantly associated with total lying time (*P* = 0.7905). The *number of lying bouts* did not differ by treatment (*P* = 0.3487), however, the number of lying bouts decreased after surgery (*P* < 0.0001), independent of treatment group (*P* = 0.4773). The *average length of a lying bout* was inversely related to the change in the number of lying bouts. It increased post-surgery (*P* < 0.0001), independently of treatment (*P* = 0.7137). There were no baseline differences between treatments (*P* = 0.1798). The *maximal length of lying bouts* did not differ between treatments (*P* = 0.1572) or sampling times (*P* = 0.0992). Interaction between predictors was not detected (*P* = 0.7926).

**Table 2.** Parameters of lying behavior before and after the surgery in the treatment groups (Mean ± SE; model-based estimates)

Behavioural parameter	Treatment	Pre-surgery	Post-surgery
	Control	444 ± 66.1	458 ± 66.1
**Total lying time (min)**	Laparoscopy	541 ± 73.9	531 ± 73.9
	Semi-laparoscopy	357 ± 80.9	289 ± 80.9
	Control	10.8 ± 1.6^a^	7.1 ± 1.6^b^
**Number of lying bouts**	Laparoscopy	14.6 ± 1.8^a^	8.3 ± 1.8^b^
	Semi-laparoscopy	12.1 ± 1.9^a^	5.4 ± 1.9^b^
	Control	41.5 ± 6.6^a^	65.2 ± 6.6^b^
**Mean length of lying bouts (min)**	Laparoscopy	40.0 ± 7.4^a^	75.3 ± 7.4^b^
	Semi-laparoscopy	28.5 ± 8.1^a^	57.9 ± 8.1^b^
	Control	110 ± 19.1	137 ± 19.1
**Maximal length of lying bouts (min)**	Laparoscopy	116 ± 21.4	149 ± 21.4
	Semi-laparoscopy	62 ± 23.4	118 ± 23.4

Different superscripts within a row indicate significant differences between pre- and post-surgery (*P* < 0.05).

## Discussion

To our knowledge, this is the first study that compared the stress associated with the L or SL technique for ET using non-invasive measures such as salivary cortisol and lying behavior. Even routine handling procedures can cause stress to the sheep (Hargreaves and Hutson, 1990), therefore, studying the pain or distress caused by ART to reduce those adverse effects is essential (Clark et al., 2006). Distress from isolation (Cockram et al., 1994) and fasting (MacKenzie et al., 1975) is not always avoidable before operations to monitor the animals and protect them from intraoperative complications. Despite the animals being separated into individual pens 2 d before the procedures in our study, they were allowed visual, tactile, and vocal contact with each other. Consequently, behavioral measures and cortisol concentrations before the trial were similar to those found normal for sheep (Pierzchała et al., 1985; ­Cockram et al., 1994).

Assisted reproduction technologies usually cause stress to the animal, even if it is a nonsurgical or minimally invasive (laparoscopic) technique. Elevation in plasma cortisol levels is expected after cervical and laparoscopic AI (Martin et al., 1981; Houdeau et al., 2002). In our study, saliva cortisol levels in the L group were higher in samples taken 1 and 2 h after the procedure, and 2 h after the procedure in the control, compared to the pre-24-h and post-24-h levels. The SL group did not show a significant increase at either time. No differences were found across groups, revealing that minimal invasive L or SL ET techniques did not cause higher stress for ewes than the handling procedure itself. Although groups showed elevated cortisol levels around the real or sham operations, Khalid et al. (1998) observed no difference in cortisol levels between ewes that received laparoscopic and transcervical AI and those that were just placed into the laparoscopic cradle. Similar to our results, these findings indicate that AI, as an ART procedure, did not cause higher stress than handling animals around the procedure (Khalid et al., 1998). When monitoring stress levels during embryo collection, the serum cortisol concentration increased more with a surgical technique compared to the transcervical method (Santos et al., 2020). However, the cortisol concentration returned to normal levels 3 h after laparotomy, while the cortisol level decreased slowly after the transcervical technique. They concluded, therefore, that the transcervical technique is less invasive. Still, the cervical manipulation has a negative effect on the ewes, even though there was anesthesia for the transcervical procedure; they still had a stress response to the manipulation of the cervix (Santos et al., 2020). Based on our results, the saliva cortisol concentration returned to the initial level 24 h after the procedures, showing that stress was eliminated within 1 d.

Although total lying time did not differ across groups before or after the treatment in our study, the mean length of lying bouts was longer in all groups during the 24-h post-surgery period compared to the pre-surgery day. Anesthesia can be one of the reasons for this phenomenon. Although no differences were found between groups, indicating that neither L nor SL techniques caused higher stress to ewes compared to the control group, the pain relief provided by these procedures was appropriate. After painful operations, lying time is often reduced, and abnormal postures and hunched standing behavior increase (Faure et al., 2017). However, appropriate pain relief can decrease pain and avoidance behavior (Fisher, 2011; Small et al., 2021). Our results agree with these findings, as the mean length of lying bouts was longer, and there were fewer lying bouts after the procedures, compared to the pre-surgery period. Another explanation for the more agitated behavior before the operation could be the effects of hunger and separation into individual pens, even though the animals had visual and vocal contact. This is a limitation of our study that we could not correctly identify the effects of hunger and separation. Further work with a higher sample size is needed to study this phenomenon.

In conclusion, our results show that with appropriate anesthesia and pain relief, L and SL ET procedures did not cause considerable stress to the animals. Moreover, animals in the SL group seemed to exhibit a lower stress response. Both methods are suitable for ET when applied “lege artis” as described in veterinary science; they are adequate in connection with the welfare concept and valuable to enhance breeding goals and shorten genetic intervals in sheep breeding. More frequent saliva sampling may be necessary during the post-surgery period to evaluate the peak level of postoperative stress and identify the most critical period for animal welfare.

## Abbreviations:

AI: artificial insemination
ART: assisted reproductive technologies
ET: embryo transfer
L: laparoscopic
MOET: multiple ovulation and embryo transfer
SL: semi-laparoscopic
